# The Influence of Mutual Assistance of Construction Workers with Different Personality Traits on Team Safety

**DOI:** 10.1155/2022/1396368

**Published:** 2022-09-16

**Authors:** Keqing Li, Ting-Kwei Wang, Anyuan Yu, Jieh-Haur Chen

**Affiliations:** ^1^School of Management Science and Real Estate, Chongqing University, Chongqing 400045, China; ^2^Department of Civil Engineering, National Kaohsiung University of Science and Technology, Kaohsiung 80778, Taiwan; ^3^Department of Civil Engineering, National Central University, Taoyuan 32001, Taiwan

## Abstract

Construction workers' unsafe behaviors are closely related to construction safety performance. Most existing studies on construction workers' personality traits and safety behaviors have ignored the flexibility of worker mix at construction sites, the dynamics of workers' behaviors, and the complexity of environmental risks at construction sites. Based on the cognitive process of construction workers' safety behaviors and from the perspective of personality traits, this research establishes an agent-based model of steelworkers' mutual assistance behavior. The AnyLogic platform is adopted to show emerging phenomena in complex problems. Through simulation experiments, the optimized configuration of construction team members under different risk environments can be obtained. This research is conducive to project managers to understand the influence of construction workers' mutual assistance on team safety, assess workers' potential for safe work before recruitment, and carry out active safety management from the source instead of looking for the cause of the accident afterward, making safety management theory more realistic and dynamic.

## 1. Introduction

According to statistics, the construction industry employs approximately 6%–10% of the labor force but accounts for 20%–40% of occupational fatal accidents [1]. The frequent occurrence of accidents makes the safety problem of building construction very serious. Researchers [2–4] analyzed the relevant data on accidents and found that the unsafe behavior of the construction personnel was the fuse for the accident. Even in the same situation, construction workers will have different behaviors. This is because, besides being affected by environmental factors, workers' behavior is also affected by personal characteristics. Many studies [5, 6] have identified personality as one factor that significantly affects workers' safety performance. Personality can also be used to explain and predict human behavior and job performance [7]. Florez and Cortissoz [8, 9] show that workgroups with similar personalities can speed up project progress. Also, personality has been proven to be related to risk perception [10], risk propensity [10], risk preference [11], unsafe behavior [11], and social behavior [12]. Although researchers have explored the relationship between workers' unsafe behaviors and personality traits, existing studies hardly consider the dynamic effects of personality traits on worker behavior and interactions between workers and the external environment.

Complex construction tasks exist on the construction site. Some traditional research methods, such as the field observation method [13] and qualitative analysis method [14], can only analyze the static state of the project but cannot capture the processes of on-site dynamic changes. Therefore, it is necessary to investigate methods that can capture the nature of dynamic site changes as well as the impacts of different personality traits.

Computer simulation technology provides a good way to solve complex systemic problems [15]. By reproducing relevant scenarios in the real world and setting parameter ranges in simulation experiments to observe changes at the macro level, the shortcomings of traditional analysis methods such as limited data, excessive interference factors, and difficulty in controlling variables [16] can be effectively avoided. Agent-based modeling (ABM) is a classic research method to explore changes in macroscopic results caused by microinteractions [17]. Aiming at the workers in the construction team and considering the mutual assistance of on-site workers and workers and the interactions with the external environment, an agent model is established in this study based on the cognitive process of behavior.

## 2. Literature Review

### 2.1. Big Five Personality Traits and Behaviors

In the construction industry, many researchers studied the factors that influence individual unsafe behaviors from the perspective of psychology. Neal and Griffin [18] proposed that personality traits affect safety behaviors and can affect safety results. Lingard and Rowlinson [19] found that there are almost no workplaces where personality does not affect work-related behaviors. Since accidents are caused by a series of events and the agents of these events are a person. Therefore, it is vital to understand the relationship between the personality characteristics of people and the high incidence of human error accidents on construction sites [20]. The Big Five personality traits, proposed by McCrae and Costa [21], are the version accepted by most psychologists, including five dimensions of extraversion, agreeableness, conscientiousness, neuroticism, and openness. In interviews, self-descriptions and observations, as well as a wide range of participants of different ages and different cultures, showed consistency [22].

Many researchers have studied the correlation between Big Five personality traits and the unsafe behavior of construction workers. Geller [23] took construction workers as the research object and explored the correlation between the five characteristics of the Big Five personality traits and unsafe behaviors through a questionnaire survey. Similarly, Clarke [24] also found that workers' accident tendency is closely related to their personality traits. Although an individual's personality traits do not directly determine whether unsafe behaviors occur, they can have an important impact on the main actions in the process of unsafe cognition. As far as risk perception is concerned, different individuals have different perceptions of risks. Different individuals have different cognitions and understandings of the origin of different risks, the composition of risks, and the severity of risks [25].

Chauvin et al. [26] found that the environment faced by construction workers is complex and changeable when performing work tasks. When making risk decisions, they are often affected by personality factors. Myers et al. [27] found that the perception of risk is closely related to individual differences based on research. The risk assessment also involves the individual's risk tolerance. Risk tolerance is related to the number of risks, the qualitative characteristics of the hazards, the perceived benefits of risks, and personal acceptability. Faced with the same risk situation, everyone's risk tolerance is different. Risk tolerance is affected by factors such as personal characteristics, expected consequences, and safety culture [28]. Thanki [29] found a correlation between personality traits and risk tolerance. Bye and Lamvik [30] also proposed that personality traits are related to risk tolerance.

### 2.2. The Cognitive Process of Safe Behavior

Many researchers found that the unsafe behaviors of construction workers mainly caused accidents. For the unsafe behavior of construction workers, the mechanism of unsafe behavior is the sorting out of the influencing factors of unsafe behavior and the construction of interrelationship to clarify the position and role of each influencing factor in the chain of unsafe behavior, which is a further deepening based on the analysis of influencing factors. With the in-depth study of psychology and social cognitive processes, Fang et al. [31] began to explain the mechanism of unsafe behavior from the perspective of safety cognition. Goh et al. [32] borrowed from the theory of planned behavior [33] to analyze and believes that unsafe behavior is the result of rational decision-making by construction workers. Chi et al. [34] pointed out that workers' unsafe behaviors are misjudgments or wrong decisions made in the cognitive process. From a physiological point of view, construction workers' cognitive status can also be assessed by valence, arousal, and dominance index in the valence-arousal-dominance (VAD) model [35], which can be measured by electroencephalography (EEG) [36]. By analyzing the three broadly influential cognitive models: Rasmussen's step-ladder model [37], Wickens et al.'s model of human information processing [38], and the IDAC model [39], Fang et al. [31] summarized the cognitive process of construction workers' unsafe behaviors into five stages: discovering information, understanding information, thinking and responding, choosing a response, and implementing the response. Among them, the failure of the choice response is the most important cause of unsafe behavior. Ye et al. [40] sorted out the influencing factors in the cognitive process and discussed the impact of the failure of the cognitive process on construction workers' unsafe behavior from the individual and the environment. The research summarizes the cognitive process into four stages: obtaining information, understanding information, choosing response, and taking action. Although there are differences in the details involved, these models all emphasize risk perception, risk assessment, and decision-making.

### 2.3. Workers' Mutual Assistance and Construction Safety

With the continuous improvement and progress of various mechanisms in human society, the emotions and instincts of cooperation and mutual assistance between individuals have become increasingly mature [41]. The behavior of mutual assistance is very important to the work and life of the individual. Herman [42] believes that mutual assistance behavior is when others have certain needs, the behavior for satisfying the real needs of others. Anderson and Williams [43] believe that helping others deal with the problems encountered in work and life, that is, the behavior of colleagues in favor of others is a mutual assistance behavior.

In the construction and production activities, the team is not only closely related to the equipment, machinery, tools, and materials required in the construction and production activities but also the most basic organization that implements the various rules and regulations, construction technology, and on-site management activities in the construction enterprise. In the construction team, most of the construction workers come from the same place. They are not only the relationship of colleagues at work but also the relationship of friends in life. No matter in life or work, construction workers will care about their workers, help and cooperate, and strengthen emotional exchanges in daily activities. Mutual help behavior not only helps team members better establish interpersonal relationships and better adapt to the surrounding environment but also has important significance in completing construction tasks safely and improving the overall performance of the team.

In the field of construction safety, many researchers have researched the mutual assistance behavior of construction workers. Liang et al. [44] believe that workers often surpass team leaders and managers and have a more social influence on workers. Some researchers [45–47] regard worker mutual assistance as a dimension to evaluate the safety atmosphere of construction and verify the relationship between the safety mutual assistance of workers and workers and the safety atmosphere of the organization. Burt et al. [46] have further realized that workers who care about the safety of their colleagues play an important role in improving safety performance. The active care of workers may overcome (or supplement) the need for management to continuously monitor safety-related behaviors. Workers help ensure the safety of their colleagues by taking on this responsibility. Suppose the dominant attitude of each worker is to care about each other, and each worker actively identifies hazards and reminds workers. In that case, the safety performance of the team will be improved. Osama Jannadi et al.'s [48] research also shows that mutual safety assistance between colleagues positively impacts safety performance.

Safety mutual help between workers is mostly in the form of communication, such as reminding workers to abide by safety rules, sharing hazard information with workers, and discussing past accidents and safety improvement measures [45, 49]. These exchanges and mutual help will not only bring about the flow of information and knowledge and other organizational resources but also positively impact the members of the organization. Knowledge and experience sharing among colleagues can better promote performance [50]. Through safe and mutual help exchanges with workers, construction workers not only make it easier for construction workers to master the operating methods of the tools and machinery used but also avoid unsafe behaviors to the greatest extent.

## 3. Research Gap

Existing studies have explored the correlation between personality traits and the work behavior of construction workers through questionnaires, focusing on the psychological factors of the agents of construction activities. However, each worker's behavior is not the direct result of a factor but rather a combination of personal characteristics, mutual assistance with others, and interaction with the external environment. In a site environment with complex construction tasks, the variability of behavior caused by different personality traits, the interactivity caused by mutual assistance and cooperation with workers, and the dynamism caused by real-time adjustment of their behavior according to site changes make the interactive behavior of individual construction personnel at the individual level lead to changes at the project level.

Thus, traditional research methods, such as the field observation method and qualitative analysis, are only possible to analyze the project situation in a static state but not to capture the dynamic changing processes. In addition, although these studies can find universal rules and provide certain guiding significance, they ignore the composition of workers' personality traits, the interactions between workers and the external environment, and the influence of construction workers' mutual assistance on team safety. Furthermore, those studies do not consider the degree of environmental risk on the construction site.

## 4. Research Methodology

### 4.1. Overview

Due to the complexity of the construction tasks, characteristics of the individuals, and the changing environment, it is more difficult to use traditional methods to study the behavior changes of construction workers and their dynamic effects [51]. Using computer simulation technology to study the behavioral activities of interaction in building construction can actively change the parameters for control, avoid the interference of unrelated external factors on the experimental results, and achieve real experimental and control effects. It can also create a new research space for construction safety-related behaviors of construction workers from the perspective of technical methods [16].

In this study, firstly, based on the findings of existing literature, the relationship between the five personality traits in the Big Five personality theory and the key parameters (risk perception, risk tolerance) in the cognitive process of unsafe behavior of construction workers was synthesized. Then, based on the workers' key parameter attributes and their external environment, whether they perform unsafe behaviors or not is determined. Finally, while the cognitive process of workers' unsafe behaviors occurs, the possibility of cooperation and mutual assistance between workers and their surrounding colleagues is considered to explore the impact of individual behaviors on the overall team safety.

This study adopts an ABM approach to solve the problem of inconvenient control of variables such as external environmental factors and an individual's actual situation when personality trait factors are involved. A variety of combinations of workers with different personality traits are realized by dynamically adjusting the relevant parameters in order to explore the different combinations of workers' mutual assistance on the overall unsafe team behavior. This study simulates the impacts of safety and mutual assistance of workers with different personality traits on team safety and provides an optimized combination plan for team workers.

### 4.2. Model Framework

**Figure 1:**
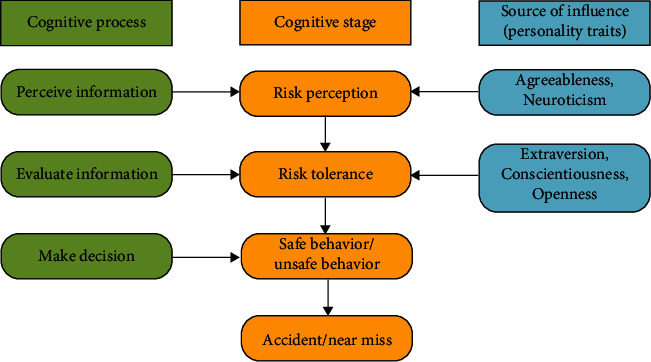
Framework of decision-making in the cognitive process of unsafe behavior of construction workers.

In recent years, research on theoretical models of cognition has matured and improved, providing a strong foundation for the scientific study and explanation of individual cognitive processes in many fields. The cognitive process of construction workers' unsafe behavior can be divided into three stages: perceiving information, evaluating information, and making decisions [52]. In the stage of perceiving information, workers perceive the risks from external information. This means the subjective judgment and assessment of the current danger when the individual is in an environment where danger may occur [53]. The risk level perceived by an individual is not only related to the true risk level [54], but risk perception is also affected by personal beliefs, attitudes, judgments, and feelings [55]. In studies on the Big Five personality traits and individual perceived risk, perceived risk is influenced by agreeableness [54] and neuroticism [26]. According to Sjöberg [54] and Chauvin et al. [26], the more neurotic and agreeableness of an individual, the more risks they feel. Individuals with high agreeableness scores may prefer safer solutions or alternatives to reduce their discomfort [56]. During the evaluation information phase, construction workers consider the level of acceptance of risk to assess the level of injury and benefit from the behavior. The risk evaluation process can be determined by comparing the perceived risk with the risk tolerance the individual can bear. Hunter [57] defines risk tolerance as “The amount of risk that an individual is willing to accept when pursuing a certain goal.” According to this definition, it can be found that risk tolerance includes two aspects: subjectivity (the degree that an individual can tolerate) and goal (total risk). Wang et al. [58] verified that the psychological characteristics of construction workers significantly impact risk tolerance through questionnaires and structural equation models. Individuals' acceptance of risk is related to their extroversion [30], openness [29], and due conscientiousness [28]. In the decision-making phase, workers make judgments based on a combination of information from the first two phases as well as physiological and skill factors. The first two cognitive stages, perceived and evaluated information, are important for the safety of construction workers. Therefore, risk perception and risk tolerance in the two stages will be selected as key parameters in the model, and the influence of personality traits on these two parameters will be investigated. The framework of decision-making in the cognitive process of unsafe behavior of construction workers is shown in Figure 1.

### 4.3. Agent-Based Model Development

Establish an agent-based model according to the cognitive process of safe behavior, which is composed of (1) a description of the environment and the agent (2) defining the mutual assistance rules between the agent and the interaction rules between the agent and the external environment (3) model validation [59]. Each of these model components is explained in detail below.

#### 4.3.1. Defining Environment and Agent

**Figure 2:**
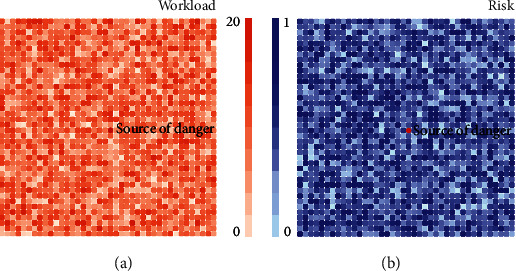
Virtual construction site: (a) workload and (b) risk level view (generated by AnyLogic software).

In the agent-based model, the virtual construction site environment is set according to the grid form proposed by Lu et al. [60]. The area is set to 40 ∗ 40 (each grid represents 1 m^2^ of space); the task volume is included in the site (range 0–20); and the risk level (range 0–1) has two parameters. Different grids have different tasks and risk levels as shown in Figure 2. Among them, parameters 1–9 are the initial parameters of the environment (1–2) and the agents (3–9) that need to be set when the model is constructed and are mainly set according to existing studies [44, 61]. Parameters 10–21 are process parameters calculated when the model is running, and the calculation method is executed according to the define interaction rules.

Because steelworkers have the highest occupational disease and injury rate [62], the workers in the model are set as steelworkers. Considering that the death rate of tower crane-related accidents is relatively serious and the location is relatively fixed, more importantly, the tower crane is easy to be noticed by colleagues' safety warnings and avoid accidents [63]. So the hazard source is set as a tower crane and placed in the center of the site.

Steelworkers are the main agents of the model, and each worker has the following state variables: ID number, Big Five personality traits (extraversion, conscientiousness, agreeableness, neuroticism, and openness), risk perception, risk tolerance, and unsafe behavior. Based on the size of the construction team, the number of workers on site is set to 20. Before each simulation model runs, the grid unit's risk value and task amount are assigned different values to simulate different construction situations. The five personality traits of construction workers are assigned different values, representing their heterogeneous attributes. Among them, different personality traits will affect the behavior and decision-making in performing tasks. Based on the statistical research of Schmitt et al. [61], the distribution of personality traits in each dimension follows the normal distribution, and the model sets its range from 0 to 1. To exclude extreme traits, the worker's traits range from 0.1 to 0.9, which is set to obey the normal distribution of *N* (0.5, 0.13) through analysis. The on-site environmental risks and on-site tasks are set to a medium level that obeys the triangular distribution [44].  Table 1 shows the parameter settings for the construction of the benchmark model (the model that has set the relevant initial parameters of the agent).

#### 4.3.2. Defining Interaction Rules

This research assumes that construction workers have two states: task-Searching and task-Executing. In task-Searching, the grid unit where the worker is currently located has no tasks, and at this time, the agent needs to move to the grid with tasks, and its state also changes from task-Searching to task-Executing. Since injuries or accidents usually occur in the task execution process rather than the task search process, this research mainly focuses on the cognitive process of safety behavior during the task execution process.

**Figure 3:**
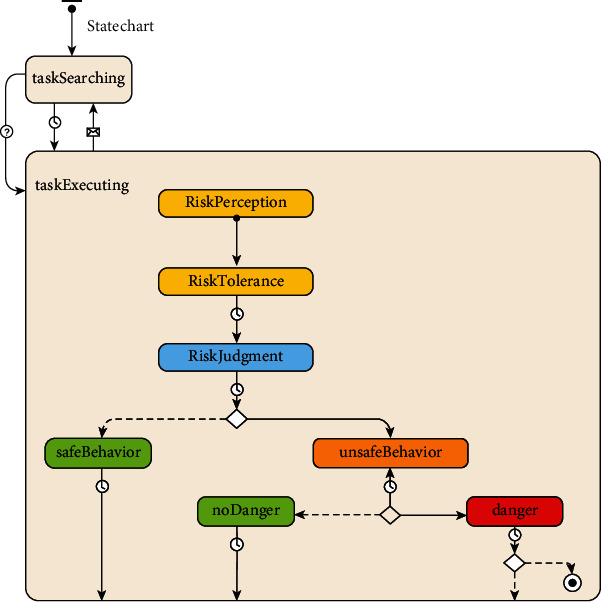
Modeling logic of workers' construction safety behavior based on the cognitive model.

*(1) The Decision-Making Rules of the Cognitive Process*. According to the conclusion of the cognitive model, workers mainly experience three stages: perception information, evaluation information, and decision-making response, in the process of cognition of unsafe behaviors, corresponding to the three states of risk perception, risk tolerance, and safe behavior or unsafe behavior. The modeling logic is shown in Figure 3.

In the stage of perceiving information, workers will make subjective judgments and assessments of hazards. Risk perception can be affected by actual risk levels [54] and individual factors [55].

Chauvin et al. [26] found that construction workers face complex and changeable environments when performing work tasks, and they are often affected by personality factors when making risky decisions. Even facing the same environmental risk, the risk perceived by one worker may be different from that of another colleague. Therefore, the risk perception in the model is determined by the actual risk value of the current environment and the personality traits of the worker. Individuals with a high level of agreeableness tend to view risk as a higher risk factor [26]. In contrast, people with high neuroticism scores are prone to anxiety and tend to be conservative in the face of risks [64]. According to Sjöberg [54] and Chauvin et al. [26], the more neurotic and agreeableness of an individual, the more risks they feel. An individual with high agreeableness scores may prefer safer solutions or alternatives to reduce their discomfort [56]. Therefore, risk perception can be determined by agreeableness and neuroticism from the perspective of personality traits. To ensure the balance of contribution, the proportion is set to 0.5. Based on the above analysis, the model can express risk perception by the following formula:

(1) $$\begin{array}{c} {\text{RP}}_{i}^{t}={\text{ER}}_{i}^{t}*\left(0.5*\frac{a_{i}}{A}+0.5*\frac{n_{i}}{N}\right), \end{array}$$

where RP_*i*_^*t*^: worker *i*'s risk perception at time *t*, ER_*i*_^*t*^: the actual risk value of worker *i*'s environment at time *t*, *a*_*i*_: the agreeableness score of workers *i*, *A*: the maximum value in the value range of agreeableness, *n*_*i*_: the neuroticism score of workers *i*, and *N*: the maximum value in the value range of neuroticism.

When workers perceive risk, they will conduct an informal assessment based on their situation, that is, assess the degree of risk that can be tolerated. Williams and Noyes [65] believe that risk tolerance is a personal assessment of risk and the corresponding comfort or discomfort to the risk. It depends on the level of confidence that controls the uncertainty of the situation. Studies have found a correlation between risk tolerance and personality traits [28–30]. Oehler and Wedlich [66] found that people with high extraversion have a higher risk tolerance, and openness positively correlates with risk-taking behavior [67]. The results of the study by Pak and Mahmood [68] show a significant negative correlation between conscientiousness and risk tolerance. Therefore, the risk tolerance set in the model is determined by the extraversion, openness, and conscientiousness of the personality traits. To ensure the balance of the distribution of traits and avoid the influence caused by a relatively high proportion of a certain trait, the proportional coefficients of the three traits are set to be 1/3 in the model. In addition to being related to their personality traits, they will also be affected by the organizational climate so that workers will adjust their behavior to conform to group norms. Moreover, the behavioral role models of coworkers can have a significant impact on individual workers and can provide individual workers with relevant information on acceptable behaviors on site [69]. Therefore, each worker will know the risk tolerance of other colleagues as a result of being affected by the safety climate of the construction team. And, due to the existence of the memory utility, the worker's risk tolerance is also determined by the average value of the risk tolerance of other workers on site in the previous time unit determined. The proposed model in this research assumes that the worker's personality traits and the influence of external colleagues have the same contribution to risk tolerance, both at 50%. Based on the above analysis, this model sets the following formula:

(2) $$\begin{array}{c} {\text{RT}}_{i}^{t}=50\text{\%}*\left(\frac{1}{3}*\frac{e_{i}}{E}+\frac{1}{3}*\frac{o_{i}}{O}-\frac{1}{3}*\frac{c_{i}}{C}\right)+50\text{\%}*\frac{1}{m}\sum_{j=1}^{m}RT_{j}^{t-1}. \end{array}$$

Here, RT_*i*_^*t*^: the risk tolerance of worker *i* at time *t*, *e*_*i*_: extraversion score of worker *i*, *E*: the maximum value in the value range of extraversion, *o*_*i*_: worker *i*'s openness score, *O*: the maximum value in the value range of openness, *c*_*i*_: worker *i*'s conscientiousness score, *C*: the maximum value in the value range of conscientiousness, *m*: the number of other workers on site, and *RT*_*j*_^*t*−1^: the risk tolerance of worker *j* at *t* − 1.

In the decision-making stage, workers make risky behavior decisions based on perceived risks and their risk tolerance. The theory of risk steady-state points out that risk perception and risk tolerance are the two main aspects that determine risk behavior [70]. Workers will compensate for the behavior if the perceived risk exceeds the internal threshold (i.e., the current risk tolerance). In the proposed model, if the perceived risk exceeds the worker's current risk tolerance, then the worker will perform safe behavior to prevent accidents. Conversely, if the perceived risk does not exceed the worker's current risk tolerance, then the worker will take unsafe behavior. Based on the above analysis, this research proposes the following formula:

(3) $$\begin{array}{c} {\text{UB}}_{i}^{t}=\left\{\begin{array}{c} 1,{\text{RP}}_{i}^{t}<{\text{RT}}_{i}^{t},\\ 0,{\text{RP}}_{i}^{t}>{\text{RT}}_{i}^{t}. \end{array}\right. \end{array}$$

Here, UB_*i*_^*t*^: worker *i*'s unsafe behavior state at time *t*, 1: take unsafe behavior, and 0: take safe actions.

Since there is no danger when taking unsafe behaviors, it may be safe and sound, which is only a near-missing event [71]. Therefore, the model assumes that when workers take unsafe behaviors, if the environment happens to be in an unsafe state, then an accident will occur; otherwise, no danger will occur, and only a near-missing accident will occur. Based on the above analysis, this model sets the following formula:

(4) $$\begin{array}{c} D_{i}^{t}=\left\{\begin{array}{cc} 1, & {\text{UB}}_{i}^{t}=1,{\text{ER}}_{i}^{t}>\text{random}.\text{uniform}\left(0,1\right),\\ 0, & \text{others} \end{array}\right. \end{array}$$

Here, *D*_*i*_^*t*^: the dangerous state of worker *i* at time *t*, ER_*i*_^*t*^: the actual risk value of worker *i*'s environment at time *t*, and random.uniform(0,1): generate a uniformly distributed floating-point number between [0, 1].

After experiencing a dangerous state, the worker's risk tolerance will change. If unsafe behaviors are taken, but no danger occurs, workers' tolerance for risks will increase and become riskier [72]. If accidents occur after unsafe behaviors, workers' risk tolerance will be weakened. That is, risk tolerance will decrease. Among them, the degree of reduction of risk tolerance is greater than the degree of increase. The purpose of the research is not to accurately measure the change value of the risk tolerance of workers after mutual assistance but to reflect the changes of different combinations of workers under different environmental risks. Therefore, after multiple simulations to observe the value range of risk tolerance, setting the reduction coefficient and the increase coefficient to 0.1 and 0.5, respectively, indicates that the change range is different. Because workers with different personality traits have different sensitivity to danger, their risk tolerance changes after the dangerous state are not the same. Therefore, after the dangerous state, the worker's risk tolerance change value is set in the model as follows:

(5) $$\begin{array}{c} {\text{RT}}_{i}^{t+1}=\left\{\begin{array}{c} {\text{RT}}_{i}^{t},{\text{UB}}_{i}^{t}=0,\\ {\text{RT}}_{i}^{t}-0.1*\left(\frac{1}{3}*\frac{e_{i}}{E}+\frac{1}{3}*\frac{o_{i}}{O}-\frac{1}{3}*\frac{c_{i}}{C}\right),{\text{UB}}_{i}^{t}=1,D_{i}^{t}=1,\\ {\text{RT}}_{i}^{t}+0.05*\left(\frac{1}{3}*\frac{e_{i}}{E}+\frac{1}{3}*\frac{o_{i}}{O}-\frac{1}{3}*\frac{c_{i}}{C}\right),{\text{UB}}_{i}^{t}=1,D_{i}^{t}=0. \end{array}\right. \end{array}$$

Here, RT_*i*_^*t*+1^: the risk tolerance of worker *i* at *t* + 1.

*(2) Decision-Making Rules for Mutual Assistance Behavior*. When construction workers are task-Executing, they will randomly engage in safe mutual assistance behaviors with surrounding workers. In the five dimensions of the Big Five personality traits, agreeableness reflects the orientation of interpersonal relationships such as trust, understanding, sympathy, and altruism [73]. Agreeableness people are good at cooperating, like to help others, and have strong empathy. Therefore, this model sets an agreeableness value as a switch for triggering safety mutual assistance behavior. According to Table 1, the agreeableness follows a normal distribution with a mean of 0.5. Therefore, it is assumed in the model that when the agreeableness of workers is higher than the average, there is an altruistic tendency and mutual assistance with other workers. Based on this, this model sets formula (6). To more accurately simulate the limitations of human perception and behavior in the actual construction environment, the range of safe mutual assistance behavior is set to workers within a radius of three meters from the workers [74].

(6) $$\begin{array}{c} \text{SHB}=\left\{\begin{array}{cc} 1, & a_{i}>0.5,\\ 0, & others. \end{array}\right. \end{array}$$

Here, SHB: safe mutual assistance behavior and *a*_*i*_: the agreeableness score of worker *i*.

Learning behavior is closely related to the process of mutual assistance. Mutual assistance from others can help individuals clarify concepts, improve problem-solving skills, and increase retention. Mutual assistance is one of the prerequisites for external learning [75]. Interaction can bring about information exchange, knowledge acquisition [76], and obvious behavior changes [77]. Mutual assistance behavior is one of the important manifestations of the interactive process. After workers undergo safe mutual assistance during construction, both participants will have new cognition and understanding of construction safety, so the feedback to the individuals of both participants is a positive behavior change rather than a negative impact. So set in the model; the two participants have a certain improvement in risk perception after mutual assistance behavior and are also more risk-conscious. Run the model multiple times to observe the value range of risk perception and set the increased value to 0.01. Based on the above analysis, this model proposes the following formula:

(7) $$\begin{array}{c} {\text{RP}}_{i}^{t+1}={\text{RP}}_{i}^{t}+0.01. \end{array}$$

#### 4.3.3. Model Verification

The purpose of model verification is to ensure that the simulation model can reasonably express the logic of the real world to solve the problem to be studied [78]. Zeigler et al. [79] divided the verification method into replicative validity (i.e., “the model matches data obtained from the real world”), predictive validity (i.e., “the model matches data before being acquired from the real world”), and construct validity (i.e., “the model truly reflects how the real world operates”). At the same time, Sargent [80] proposed that an appropriate verification method should be selected according to the purpose of the model. This paper aims to explore the role of construction workers with different personality traits on safety-related behaviors based on the cognitive mechanism of safety behaviors, rather than to make accurate predictions of safety behaviors. Therefore, the verification of this model will focus on replicative validity and construct validity.

**Figure 4:**
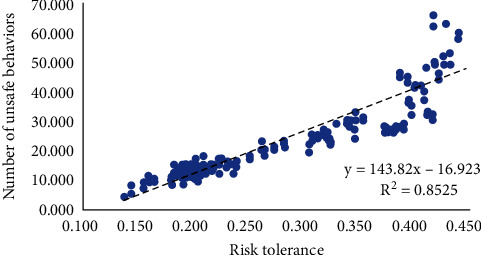
The relationship between the average risk tolerance of construction workers and the number of unsafe behaviors.

First, a qualitative consistency test of replicative validity was carried out for the model. It can be seen from Figure 4 that the overall risk tolerance level of construction workers in the benchmark model is directly proportional to the number of unsafe behaviors (the total number of unsafe behaviors taken by workers in the team from the beginning of the task to the current model time; *R*^2^ = 0.852, *p* < 0.001). That is, the higher the level of construction workers who can tolerate risks, the more unsafe behaviors may occur during the execution of the task. This result is supported by existing literature in many fields. Ji et al. [81] interviewed pilots and used questionnaires to analyze the relationship between pilots' behavioral decision-making and risk tolerance. The results showed that the higher the risk tolerance, the fewer safe behaviors. Davidson [82] found that the higher the coal miners' tolerance to risk, the greater the risk of their decision-making plans by studying the process of coal miners' final behavior selection. In construction, Ma [83] established a construct equation model, conducted empirical analysis, and found that the risk tolerance of construction workers was significantly negatively correlated with safety behavior.

**Figure 5:**
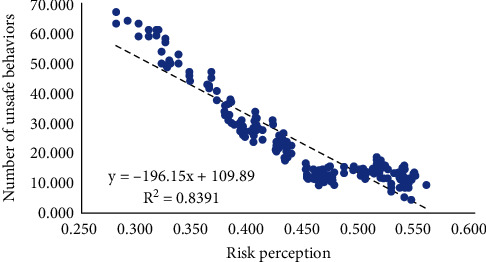
The relationship between the average risk perception level of construction workers and the number of unsafe behaviors.

The simulation results are consistent with existing studies on the relationship between risk perception and unsafe behavior. As shown in Figure 5, the unsafe behavior of construction workers will be inhibited by their risk perception level (*R*^2^ = 0.839, *p* < 0.001). Under the same level of risk environment, the more risks construction workers can perceive and the more comprehensive, the higher the probability of taking safe actions. Existing studies also support this result. The research of Xia et al. [84] found that the more risks construction workers perceive, the more they can recognize the potential dangers in the current state and thus can choose safe behaviors to prevent themselves from accidents. Huang et al. [85] found that risk perception and unsafe behavior have a significant negative correlation through a survey of front-line workers in Chinese construction projects. Xia et al. [86] found that improving the risk perception of construction workers can play a positive role in safe behavior.

To ensure the quantitative consistency of replicative validity, this study implemented the benchmark model 50 times, calculated the mean value of important indicators, and compared the results with the empirical data of previous studies. Among them, the unsafe behavior rate (formula (8)) is 0.35 (standard deviation = 0.006), which is consistent with the research results of Sa et al. [87] and Fang and Wu [88]. Both studies have found that one-third of workers are unsafe at the construction site. Also, according to Heinrich's [89] triangle rule, every major accident will cause 29 serious accidents and 300 near-missing accidents. That is, the ratio of accidents to near-missing accidents (formula (9)) is roughly 1:10. Execute the benchmark model 50 times and calculate that the ratio of accidents to near-missing events is 1:8.6, which is near the value of the triangle rule. Finally, the average accident rate is calculated and compared with the relevant accident statistics. The benchmark model is executed 50 times, and the accident rate (formula (10)) is calculated to be 3.3, which is the same as the 2016 Occupational Injury and Disease Incident Rate (3.0) published by the U.S. Bureau of Labor Statistics [90] very close. Therefore, the quantitative consistency between the simulation results and the empirical data is proved.

(8) $$\begin{array}{c} \text{unsafe behavior rate}=\frac{\text{number of unsafe behaviors}}{\text{number of un safe behaviors}+\text{number of safe behaviors}}, \end{array}$$

(9) $$\begin{array}{c} \text{ratio of accidents to near misses}=\frac{\text{number of accidents}}{\text{number of nearmissing accidents}}\text{,} \end{array}$$

(10) $$\begin{array}{c} \text{accident rate}=\frac{\text{number of accidents}}{\text{number of unsafe behaviors}+\text{number of safe behaviors}}\text{.} \end{array}$$

In terms of construct validity, this study uses three methods to enhance the construct validity of the model. First, the agent's behavioral rules and interaction rules are based on theories that have been established and verified in the social science literature (such as cognitive science theory, risk homeostasis theory, and Big Five personality traits). Secondly, the influencing factors of parameters (such as risk perception and risk tolerance) are derived from the research results of the existing literature. Finally, the initialization of model parameters refers to the common principles in existing empirical research and related construction simulation literature [52, 60].

## 5. Experiments and Results

### 5.1. Experimental Design

Based on the parameters set by the benchmark model, this study changed the risk level of the construction environment. The model kept running until the changes in each variable have stabilized (elapse of 80 model time units).

**Figure 6:**
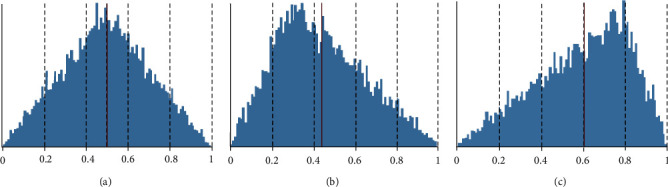
Different environmental risk levels (indicated by triangular distribution function): (a) medium risk triangular (0, 1, 0.5), (b) low risk triangular (0, 1, 0.3), and (c) high risk triangular (0, 1, 0.8).

In the experiment, the construction workers in the construction team were divided into two groups (A and B), and each group has ten workers. The five dimensions of the personality traits of the construction workers have two levels, high and low. For example, extraversion is divided into high-level extraversion (i.e., extroversion, denoted by 0.7) and low-level extroversion (i.e., introversion, denoted by 0.3). The variable traits of the two groups of workers will take a combination of high and low levels. The personality traits of other dimensions all obey the normal distribution. In the benchmark model, all traits are set to follow a normal distribution. Take the extraversion as an example. The experiment sets the parameters as shown in Table 2 to explore the relevant effects of different combinations of extraversion. Different levels of environmental risk are set by changing the mode value of the triangular distribution, as shown in Figure 6.

### 5.2. Simulation Results

**Figure 7:**
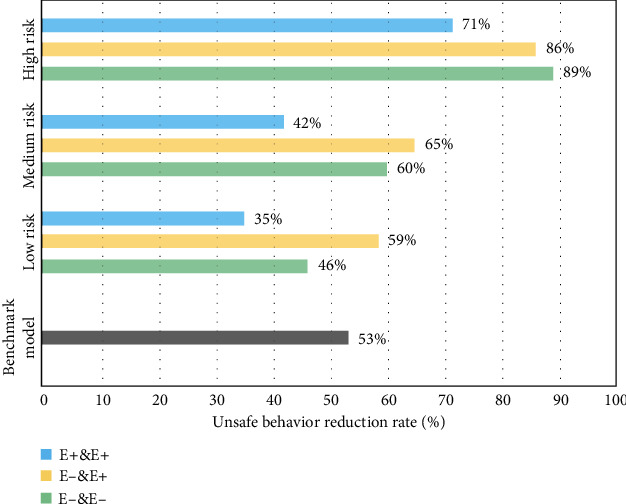
The reduction rate of unsafe behaviors of construction workers with different levels of extroversion (E) under different risk environments.

**Figure 8:**
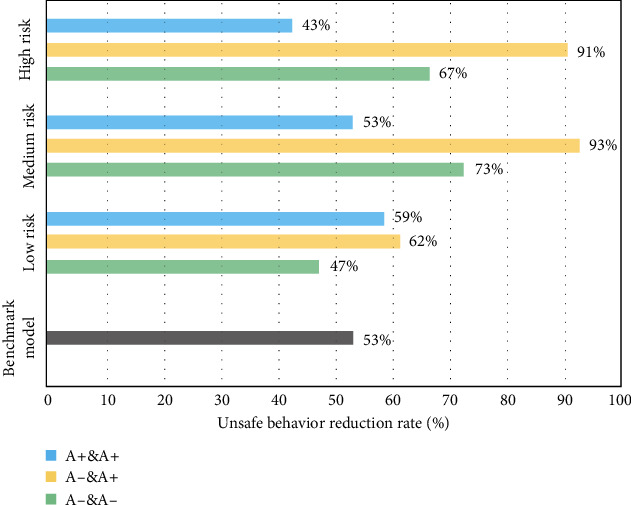
The reduction rate of unsafe behaviors of construction workers with different levels of agreeableness (A) under different risk environments.

**Figure 9:**
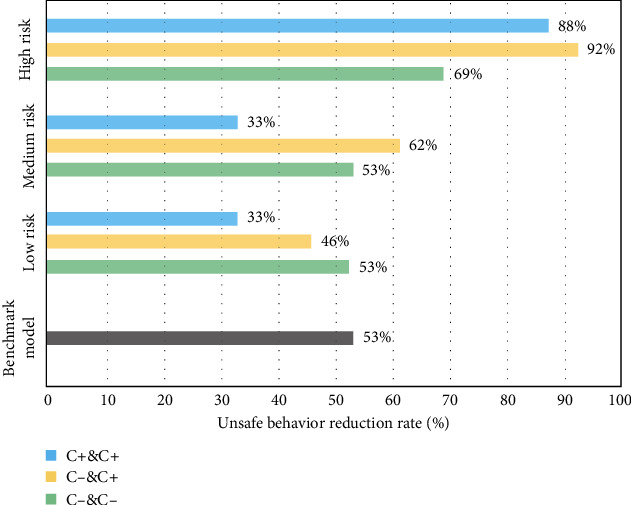
The reduction rate of unsafe behaviors of construction workers with different conscientiousness (C) levels under different risk environments.

**Figure 10:**
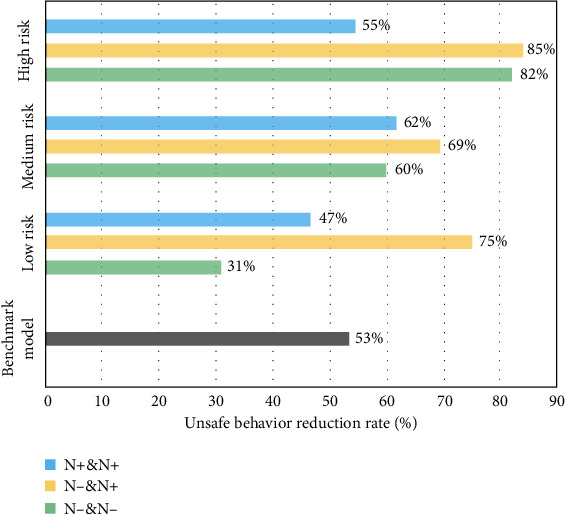
The reduction rate of unsafe behaviors of construction workers with different neuroticism (N) levels under different risk environments.

**Figure 11:**
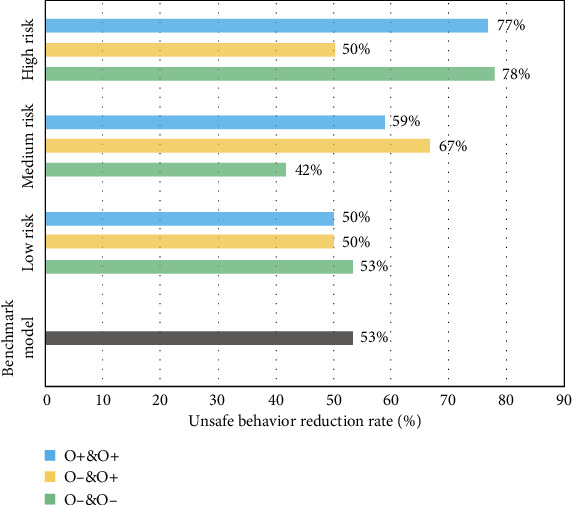
The reduction rate of unsafe behaviors of construction workers with different openness (O) levels under different risk environments.

Figures 7–11, respectively, show the combination of construction workers with different personality traits in high-, medium-, and low-risk environments. After the cognitive process of unsafe behavior and the process of mutual assistance, they reflect the change in the rate of unsafe behavior reduction on the overall level of the construction team (i.e., the ratio of the difference between the number of unsafe behaviors before and after the safe mutual assistance behavior and the number of unsafe behaviors before mutual assistance). It should be pointed out that the average unsafe behavior rate of construction workers within the construction team in the baseline model was reduced by 53% in the medium-risk environment.

It can be seen from Figure 7 that with the increase of environmental risks, the average unsafe behavior reduction rate of the three types of extraverted combination forms of team workers is getting lower. This is because the high-risk environment is relatively more dangerous and prone to accidents. The resulting warning effect will prompt workers to pay more attention to construction safety. As far as the combination is concerned, regardless of the high or low environmental risk, the team with the same number of high- and low-extroverted workers has the largest reduction rate of unsafe behavior, and the overall safety performance of the team has the best improvement effect. The team with more highly extroverted workers has the lowest reduction rate of unsafe behavior, which is caused by the characteristics of highly extroverted individuals who like to take risks and pursue excitement.

In Figure 8, as far as the combination is concerned, regardless of the high or low environmental risk, teams with the same number of agreeableness workers have the greatest reduction in unsafe behaviors. At this time, the overall safety behavior of the team has the best improvement, especially in the medium- and high-risk environments by about 90%. This may be because workers with different levels of agreeableness are more efficient in learning safety in mutual assistance behaviors, thereby reducing the occurrence of unsafe behaviors.

Figure 9 shows that with the increase in environmental risk levels, the average incidence of unsafe behaviors of the three types of conscientious combination of team workers is getting lower. This is because the environmental risk becomes higher, the greater the impact of conscientiousness; workers will carefully assess the safety situation before performing tasks and act more cautiously so that there are fewer unsafe behaviors. Especially in a high-risk environment, in teams with more highly conscientious workers or teams with the same number of high- and low-conscientious workers, the unsafe behavior of construction workers after mutual assistance is reduced by about 90%.

It can be found in Figure 10 that in terms of the combination form, regardless of the level of environmental risk, team workers with the same number of high- and low-neurotic workers have the greatest reduction in unsafe behavior, and the improvement of team safety behavior is the best, especially in the high-risk environment; it is reduced to 85%. Teams with more highly neurotic or less neurotic workers are more effective in reducing unsafe behaviors in medium to high risks but are less effective in low-risk environments. This may be due to workers' safety mentality being more relaxed and emotionally stable and will not always worry about accidents in low-risk environments.

Figure 11 shows that in a low-risk environment, the reduction rates of unsafe behaviors of workers in a combination of the three types of openness levels after mutual assistance are very small, all of which are around 50%. In a medium-risk environment, when the overall openness of the team is at the mid-to-high level, mutual assistance has the best effect on safety. While in a high-risk environment, the overall openness and consistency of the team are more important. The overall reduction rate of unsafe behaviors in teams with high or low overall openness after mutual assistance is nearly 80%, which can effectively improve team performance.

Based on the results of the above experiments, according to the idea that the higher the rate of unsafe behavior reduction after the mutual assistance of the construction workers of the team is, the better the improvement of safety performance. It can analyze the best combination of different personality traits to improve the overall safety level of the team under various levels of environmental risks as shown in Table 3.

In a low-risk environment, when the number of high and low levels of extravert, agreeable, and neurotic workers in the team is similar, and there are more low-conscientious and low-neurotic workers, the effect of improving team safety is best. In a medium-risk environment, the team has the best potential for safety performance when the team has much the same as the workers with high and low levels of each trait. In a high-risk environment, when the number of high and low levels of agreeable, conscientious, and neurotic workers in the team is almost identical and there are more low-extraversion and low-openness workers, the team's safety behavior improvement effect is best after worker safety mutual assistance. It can be found that when the number of workers with high and low levels of extraversion, agreeableness, and neuroticism is nearly equal, regardless of the level of environmental risk, mutual assistance improves the overall safety of the construction team.

## 6. Discussion and Conclusion

This paper mainly explores the change difference in the overall unsafe behavior of the workers after the safety mutual assistance of workers with different personality traits. First, based on the occurrence mechanism of unsafe behaviors of construction workers, the critical processes in the cognitive process of unsafe behaviors are extracted: risk perception, risk tolerance, and decision-making. Existing studies indicate that the Big Five personality traits affect the risk perception and risk tolerance in the process of individual unsafe behaviors and indirectly determine whether unsafe behaviors of construction workers occur.

Agent-based modeling (ABM) is used in this paper as a bottom-up and micro-to-macro classical modeling approach. At the same time, the research analyzes the relationship between construction workers and workers and the relationship between construction workers and the external construction environment to establish individual behavior rules and interaction rules that are consistent with the actual construction context. The individual worker's personality trait, an inherent attribute that cannot be easily changed later in life, determines each worker's unique outwardly expressive behavior and decision-making style. By setting up worker agents possessing different traits in the agent-based model and arranging various combinations of workers in the team, it is possible to maximize the simulation of the behavioral differences of workers with different personality traits in the real environment and measure the impact of such differences on overall team safety performance. This paper explores the optimal combination of workers with varying traits of personality in various risk environments from the perspective of psychology, providing a new way to improve the safety performance of teams in different situations and ultimately deepen the understanding of the complex system in construction.

By introducing the Big Five personality traits theory into the research of the construction industry, combined with the agent-based modeling method in the complex system theory, this paper is conducive to understanding the cognitive process of construction workers' unsafe behavior and the differences in the results of unsafe behavior from the perspective of psychology. According to the conclusions of the simulation analysis, this research proposes corresponding management measures to improve construction safety from the perspective of enterprise human resources and personnel arrangements based on personality traits and actively manages from the source to improve the level of construction safety performance.

## Figures and Tables

**Table 1:** Model parameter setting.

No.	Parameter name	Parameter meaning	Initialization value
1	riskLevel	Construction site environmental risks	Triangular (0, 0.5, 1)
2	workload	Construction site tasks	Triangular (0, 10, 20)
3	workerNum	Number of workers	20
4	id	Worker's ID	—
5	extroversion (E)	Worker's extroversion	*N* (0.5, 0.13)
6	agreeableness (A)	Worker's agreeableness	*N* (0.5, 0.13)
7	conscientiousness (C)	Worker's conscientiousness	*N* (0.5, 0.13)
8	neuroticism (N)	Worker's neuroticism	*N* (0.5, 0.13)
9	openness (O)	Worker's openness	*N* (0.5, 0.13)
10	riskPerception (RP)	Risk perception of worker	—
11	riskTolerance (RT)	Risk tolerance of worker	—
12	unsafeBehavior (UB)	Unsafe behavior of worker	—
13	safeHelpBehavior (SHB)	Mutual assistance of workers	—
14	numberOfUnsafeBehaviors	Number of worker's unsafe behaviors	—
15	numberOfSafeBehaviors	Number of worker's safe behaviors	—
16	unsafeBehaviorRate	Unsafe behavior rate	—
17	numberOfAccidents	Number of accidents	—
18	numberOfNear-missingAccidents	Number of near-missing	—
19	ratioOfAccidentsToNearMisses	Ratio of accidents to near-missing	—
20	unsafeBehaviorReductionRate	Unsafe behavior reduction rate	—
21	accidentRate	Accident rate	—

**Table 2:** Big Five personality parameter settings in the experiment (extraversion as an example).

	Group A	Group B
E	0.3	0.3
	0.3	0.7
	0.7	0.7

A	*N* (0.5, 0.16)	*N* (0.5, 0.16)

C	*N* (0.5, 0.16)	*N* (0.5, 0.16)

N	*N* (0.5, 0.16)	*N* (0.5, 0.16)

O	*N* (0.5, 0.16)	*N* (0.5, 0.16)

**Table 3:** The best combination of workers' personality traits under various levels of environmental risks to improve safety performance.

Environmental risk level	Low		Medium		High	
Group	Group A	Group B	Group A	Group B	Group A	Group B
Extroversion	High	Low	High	Low	Low	Low
Agreeableness	High	Low	High	Low	High	Low
Conscientiousness	Low	Low	High	Low	High	Low
Neuroticism	High	Low	High	Low	High	Low
Openness	Low	Low	High	Low	Low	Low

## Data Availability

The data used to support the findings of this study are included within the article.
